# Refractory Chylous Ascites Leading to Chylothorax Following Extended Right Hepatectomy for Intrahepatic Cholangiocarcinoma

**DOI:** 10.7759/cureus.73301

**Published:** 2024-11-08

**Authors:** Koshiro Morino, Yuki Morimura, Hirokazu Tanaka, Takafumi Machimoto, Tatsuo Nakagawa

**Affiliations:** 1 Department of Gastroenterological Surgery, Tenri Hospital, Tenri, JPN; 2 Department of Thoracic Surgery, Tenri Hospital, Tenri, JPN

**Keywords:** chylothorax, chylous ascites, hepatectomy, liver resection, lymphadenectomy, postoperative complications

## Abstract

Chylous ascites, a rare but severe complication of abdominal surgery, often results from lymphatic vessel damage during procedures, such as extended resection and lymphadenectomy. Although conservative management through dietary modifications and medications is the primary approach, refractory cases may lead to severe complications including nutritional deficiencies and even death. Herein, we report a case of refractory chylous ascites that progressed to chylothorax after extended right hepatectomy with lymph node dissection for intrahepatic cholangiocarcinoma. A 73-year-old woman developed chylous ascites one month postoperatively, which subsequently perforated the diaphragm and led to a massive chylothorax. Despite conservative management including fasting and drainage, surgical intervention was required to resolve the condition. This case highlights the importance of timely recognition and treatment of refractory chylous ascites after extensive liver resection.

## Introduction

Chylous ascites during abdominal surgery is generally attributed to injury to lymphatic vessels, particularly near the cisterna chyli, and can result in the accumulation of chylous fluid in the peritoneal or pleural spaces [1]. Although rare, the incidence of chylous ascites may be increasing owing to the growing frequency of extensive resections and lymphadenectomies in modern surgical practice [2]. Chylous ascites is typically identified postoperatively by the appearance of milky drainage in surgical drains and is often observed following the resumption of oral intake [3]. The majority of patients respond to conservative management, including fasting and dietary modifications [4]. However, refractory chylous ascites that is resistant to conservative therapy can result in prolonged leakage, leading to malnutrition, cachexia, and potentially fatal outcomes [5].

Despite its severity, no high-level evidence or well-established guidelines currently exist for the management of postoperative chylous ascites. This report presents a case of refractory chylous ascites that developed after extended right hepatectomy and lymphadenectomy for intrahepatic cholangiocarcinoma (ICC). The ascites eventually progressed to chylothorax, perforating the diaphragm, and required a staged surgical approach for resolution.

## Case presentation

**Figure 1:**
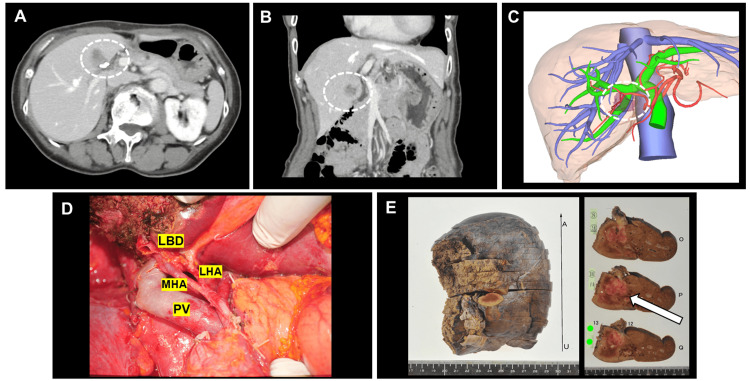
Preoperative and intraoperative images from previous surgery. (A) Axial and (B) coronal contrast-enhanced CT images and (C) 3D reconstruction showing the tumor (outlined with a dashed circle). (D) Intraoperative image after extended right hepatectomy with lymphadenectomy. (E) Resected specimen showing negative surgical margins (white arrow). PV: portal vein; LHA: left hepatic artery; MHA: middle hepatic artery; LBD: left bile duct

A 73-year-old woman with a history of chronic hepatitis C virus infection, successfully treated with antiviral therapy (sofosbuvir/ledipasvir), was followed up in a gastroenterology clinic. During routine follow-up, a 2-cm mass was detected in the hepatic hilum (S4-S5) by ultrasound, and further investigation confirmed ICC with invasion into the hepatic hilum (Figure 1, panels A-C). No findings suggestive of distant metastasis or lymph node metastasis were observed on CT, MRI, or other imaging modalities, and the lesion was deemed resectable.

**Figure 2:**
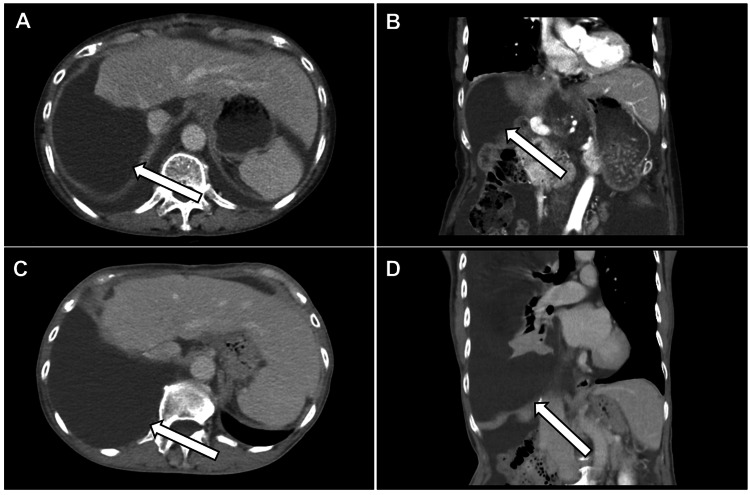
CT images of chylous ascites and chylothorax. (A) Axial and (B) coronal contrast-enhanced CT images showing chylous ascites. (C) Axial and (D) coronal contrast-enhanced CT images showing a chylothorax. Chylous ascites and chylothorax are indicated by white arrows.

Preoperative liver function tests revealed that a future liver remnant (FLR) was 33%, with an indocyanine green clearance of 0.157 and a remnant liver function coefficient (K-rem) of 0.051, indicating a borderline case for resection [6]. Portal embolization of the right portal vein was performed to achieve an FLR of 44% and a K-rem of 0.063. One month later, an extended right hepatectomy with caudate lobectomy, lymphadenectomy, and biliary reconstruction was performed (Figure 1, panel D). The patient was discharged on postoperative day 20, without any immediate complications. The pathological diagnosis was stage IVA ICC, with a tumor size of 2.5 cm, single tumor (St), expansive growth (eg), fibrous capsule (negative for invasion) (fc{-}), no septum formation (sf{-}), no serosal invasion (s0), lymph node metastasis present (n1), minimal portal vein invasion (vp1), no hepatic vein invasion (vv0), no arterial invasion (va0), no peritoneal dissemination (p0), surgical margin negative with a clearance of 5 mm (sm{-} {5 mm}), no fibrosis (f0), pathological stage T3 (pT3), four out of 17 lymph nodes positive for metastasis (N1 {4/17}), in accordance with the Sixth Edition of the Japanese Classification of Liver Cancer (Figure 1, panel E). One month after discharge, the patient developed abdominal distension and ascites. The blood laboratory results are presented in Table 1, and the computed tomography (CT) images are shown in Figure 2, panels A and B.

**Table 1:** Laboratory results at the diagnosis of chylous ascites and progression to chylothorax. Result 1: diagnosis of chylous ascites; result 2: progression to chylothorax

Test	Result 1	Result 2	Normal range
Hemoglobin	12.2 g/dL	13.8 g/dL	13.7-16.8 g/dL
Mean corpuscular volume	90.3 fL	90.4 fL	83.6-98.2 fL
Platelets	39.2×10^4^/µL	19.8×10^4^/µL	15.8-34.8×10^4^/µL
White blood cell	7.81×10^3^/µL	4.69×10^3^/µL	3.3-8.6×10^3^/µL
Total bilirubin	0.4 mg/dL	0.6 mg/dL	0.4-1.5 mg/dL
Alanine transaminase	15 U/L	30 U/L	10-42 U/L
Alkaline phosphatase	74 U/L	64 U/L	38-113 U/L
Aspartate transaminase	23 U/L	36 U/L	13-30 U/L
Lactate dehydrogenase	196 U/L	182 U/L	124-222 U/L
Blood urea nitrogen	16.4 mg/dL	29.3 mg/dL	8-20 mg/dL
Creatinine	0.6 mg/dL	0.6 mg/dL	0.7-1.1 mg/dL
Total protein	5.8 g/dL	5.1 g/dL	6.6-8.1 g/dL
Albumin	2.7 g/dL	2.4 g/dL	4.1-5.1 g/dL
Cholinesterase	128 mg/dL	116 mg/dL	142-248 mg/dL
C-reactive protein	12.02 mg/dL	<0.05 mg/dL	<0.14 mg/dL
Carcinoembryonic antigen	2.5 ng/mL	3.5 ng/mL	<5 ng/mL
Carbohydrate antigen 19-9	21.3 U/mL	20.4 U/mL	<37 U/mL

Abdominal paracentesis revealed milky chylous ascites, which was confirmed using biochemical analysis. The ascitic fluid had a triglyceride level of 1,571 mg/dL, with negative bacterial culture results and no malignant cells on cytological analysis. Conservative management was initiated, including fasting, a low-fat diet, and octreotide administration, leading to temporary improvement of the patient’s condition. Subsequently, diuretics (tolvaptan, furosemide, and spironolactone) were administered.

**Figure 3:**
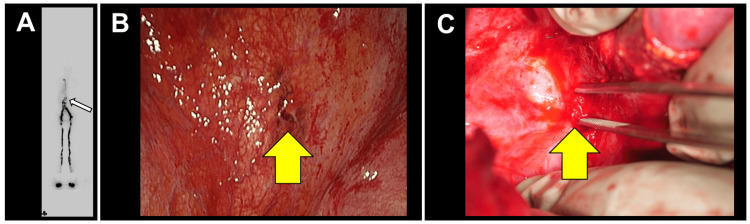
Chyle leakage evaluation and intraoperative images. (A) Lymphoscintigraphy showing no significant lymphatic leakage (white arrow) into systemic circulation. (B) Intraoperative image from the initial thoracic surgery showing diaphragmatic disruption and chyle leakage (yellow arrow) within the thoracic cavity. (C) Intraoperative image showing chyle leakage (yellow arrow) from the hepatic hilum lymphatic system.

Three months later, the patient developed a significant right pleural effusion (Figure 2, panels C and D). Thoracentesis yielded 2 L of milky fluid, confirming a diagnosis of chylothorax. The pleural fluid had a triglyceride level of 483 mg/dL, negative bacterial culture results, and no malignant cells on cytological analysis. Imaging suggested that chylous fluid originating from the abdominal cavity perforated the diaphragm. The blood tests performed at that time revealed a worsened nutritional status compared with that performed at the initial detection of chylous ascites (Table 1). Conservative measures including total parenteral nutrition and chest drainage were employed; however, persistent drainage exceeding 500 mL/day over two weeks suggested treatment resistance. Lymphoscintigraphy showed the absence of leakage from the thoracic duct or systemic lymphatic vessels, indicating that the lymphatic dissection site in the hepatic hilum was the source of leakage (Figure 3, panel A). Percutaneous puncture lymphangiography for lymphatic leakage at the hepatic hilum was considered; however, this option was not pursued as there are no physicians at our institution qualified to perform the procedure.

Given the patient’s deteriorating condition, a two-stage surgical approach was planned as described below. (1) Thoracoscopic diaphragmatic repair: a diaphragmatic communication was identified (Figure 3, panel B) and closed with fibrin glue (Beriplast) and a tissue sealant (Neoveil). An abdominal drain was placed to relieve the pressure. (2) Abdominal re-exploration: one week later, open abdominal surgery was performed to identify and ligate the leaking lymphatic vessels near the hepatic hilum (Figure 3, panel C). Similar to the thoracic procedure, fibrin glue and sealants were applied to stop leakage. Prior to both procedures, the patient ingested 250 cc of milk to identify the site of chyle leak.

**Figure 4:**
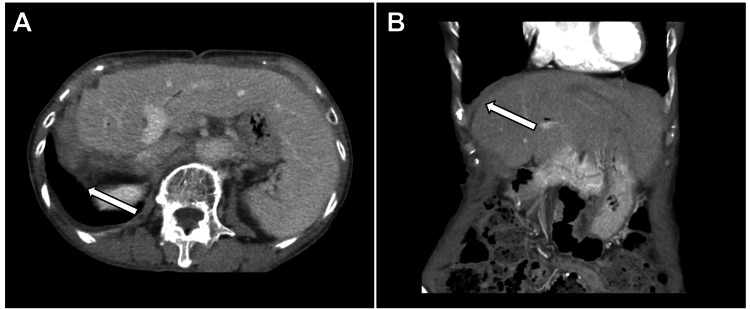
Post-treatment CT images. One month postdischarge contrast-enhanced CT showing the absence of pleural or peritoneal effusion (white arrow) in (A) axial and (B) coronal views.

The patient recovered uneventfully after the procedure. The thoracic drain was removed after pleurodesis on postoperative day five, whereas the abdominal drain was removed after one week. Oral intake was resumed two weeks later, and the patient was discharged 22 days after the second surgery. A follow-up CT scan one-month postdischarge showed the absence of recurrence of pleural or peritoneal effusion, indicating successful resolution of the chylous ascites (Figure 4, panels A and B).

## Discussion

This report describes a rare case of chylous ascites that progressed to massive chylothorax through diaphragmatic perforation, following extended hepatectomy with lymphadenectomy. This study documents chylous ascites that progressed to chylothorax through this mechanism, highlighting the unique challenges posed by refractory chylous ascites during abdominal surgery.

Chylous ascites is a considerable postoperative complication given the increasing prevalence of extensive resection and lymphadenectomy [2]. The condition is characterized by the presence of milky fluid in the postoperative abdominal drainage with elevated triglyceride levels (diagnosed as chylous ascites when >110 mg/dL) [3]. Typically, this results from surgical trauma to the main thoracic duct, its branches, or the lymph nodes. Prolonged chylous ascites can lead to significant nutritional deficiencies owing to the loss of proteins, lymphocytes, and immunoglobulins [5]. However, the absence of established management guidelines, particularly in refractory cases, presents formidable challenges that may lead to life-threatening outcomes if not promptly addressed.

Chylothorax can be classified as traumatic or non-traumatic, with the former further subdivided into iatrogenic and non-iatrogenic etiologies [7]. Iatrogenic cases predominantly result from thoracic duct injuries during surgery, with the highest incidence reported during esophagectomy (2%-4%) [8], followed by lung resection (0.5%-2%) [9]. Non-iatrogenic causes include both penetrating and blunt chest traumas. However, none of these factors lead to chylous ascites following liver resection. Although chylous ascites rarely occurs after liver resection, the development of chylothorax in this context is a previously unreported complication [10]. The primary cause of the diaphragmatic perforation is believed to be increased intra-abdominal pressure. However, there are no prior reports of diaphragmatic perforation resulting solely from increased intra-abdominal pressure. Although there was no tumor invasion at the time of the initial surgery, it is possible that thermal injury to the diaphragm during mobilization of the right hepatic lobe led to localized weakening in certain areas.

Conservative management is the first-line treatment for chylous ascites and typically involves dietary modification, fasting, and nutritional support [4,5]. Initial management should focus on monitoring the volume of chyle drainage, with outputs below 500 mL/day generally considered low output [11]. In patients with low output, a low-fat, high-protein diet is recommended, with medium-chain triglycerides (MCT) as the primary fat source, as MCTs are primarily absorbed through the portal circulation rather than the intestinal lymphatics, reducing stress on damaged lymphatic vessels and supporting healing [5]. However, a unanimous consensus on the MCT diet’s effectiveness has not yet been reached.

Pharmacological interventions, particularly octreotide, a somatostatin analog, may also be administered to reduce lymphatic flow by decreasing gastrointestinal and splanchnic blood flow [12]. Previous studies have demonstrated the efficacy of octreotide in combination with dietary management to reduce the volume and duration of chylous ascites. For refractory patients, lymphangiography with lymphatic embolization is considered a valuable option, with success rates of up to 70% in patients with chylothorax [13,14]. However, peripheral lymphatic leaks at the hepatic hilum or mesentery are not typically visualized through lymphatic flow imaging in the systemic circulation [2]. There are only a few reports of lymphangiography for hepatic hilum lymphatic leaks via percutaneous puncture of the hepatic hilum [15].

Surgical intervention becomes necessary when conservative measures fail, although it carries significant risks. While a standardized approach to surgical management has not been established, the basic principle involves identifying and ligating the leaking lymphatic vessels, as was required in our patient. The chyle leak can be localized by administering fatty foods or creams preoperatively or intraoperatively. Additionally, recent reports have shown successful outcomes in resolving chyle leaks by supplementing vessel ligation with the application of polyglycolic acid felt and fibrin glue, which was also utilized in our patient [16,17].

## Conclusions

This is a rare case of refractory chylous ascites progressing to chylothorax after extensive hepatic surgery with lymphadenectomy. Although conservative management is the first line of treatment for chylous ascites, timely surgical intervention is crucial to prevent life-threatening complications in refractory cases. Further studies are required to establish standardized guidelines for managing such complex cases.
